# Intracellular hemin is a potent inhibitor of the voltage-gated potassium channel Kv10.1

**DOI:** 10.1038/s41598-022-18975-2

**Published:** 2022-08-27

**Authors:** Nirakar Sahoo, Kefan Yang, Ina Coburger, Alisa Bernert, Sandip M. Swain, Guido Gessner, Reinhard Kappl, Toni Kühl, Diana Imhof, Toshinori Hoshi, Roland Schönherr, Stefan H. Heinemann

**Affiliations:** 1 Department of Biophysics, Center for Molecular Biomedicine, Friedrich Schiller University Jena & Jena University Hospital, Hans-Knöll-Str. 2, 07745 Jena, Germany; 2 Department of Biology, The University of Texas Rio Grande Valley, 1201 West University Drive, Edinburg, TX 78539 USA; 3 Institute of Biophysics, Saarland University, 66421 Homburg, Germany; 4 Pharmaceutical Biochemistry and Bioanalytics, Pharmaceutical Institute, University of Bonn, An der Immenburg 4, 53121 Bonn, Germany; 5 Department of Physiology, University of Pennsylvania, Philadelphia, PA 19104-6085 USA; 6 Present Address: Department of Medicine, Duke University, Durham, NC 27710 USA

**Keywords:** Biophysics, Physiology

## Abstract

Heme, an iron-protoporphyrin IX complex, is a cofactor bound to various hemoproteins and supports a broad range of functions, such as electron transfer, oxygen transport, signal transduction, and drug metabolism. In recent years, there has been a growing recognition of heme as a non-genomic modulator of ion channel functions. Here, we show that intracellular free heme and hemin modulate human ether à go-go (hEAG1, Kv10.1) voltage-gated potassium channels. Application of hemin to the intracellular side potently inhibits Kv10.1 channels with an *IC*_50_ of about 4 nM under ambient and 63 nM under reducing conditions in a weakly voltage-dependent manner, favoring inhibition at resting potential. Functional studies on channel mutants and biochemical analysis of synthetic and recombinant channel fragments identified a heme-binding motif CxHx_8_H in the C-linker region of the Kv10.1 C terminus, with cysteine 541 and histidines 543 and 552 being important for hemin binding. Binding of hemin to the C linker may induce a conformational constraint that interferes with channel gating. Our results demonstrate that heme and hemin are endogenous modulators of Kv10.1 channels and could be exploited to modulate Kv10.1-mediated cellular functions.

The human ether à go-go channel 1 (hEAG1, Kv10.1) is a voltage-gated delayed rectifier potassium (K^+^) channel encoded by the *KCNH*1 gene. It is the founding member of the EAG family, which also includes Elk and Erg channels. While the human Erg1 (hERG1, Kv11.1) is critically involved in cardiac excitability, Kv10.1 is primarily found in the vertebrate nervous system, including the neocortex, olfactory bulb, hippocampus, retinal ganglion, photoreceptors, cochlear spiral ligament, and skeletal muscle^1–4^. The physiological importance of Kv10.1 channels in the nervous system is readily evidenced by the hereditary mutations that cause human diseases such as Zimmermann-Laband and Temple-Baraitser syndrome. The respective patients suffer from severe neurological disorders ranging from epilepsy, marked hypotonia in infancy, focal tonic–clonic seizures, severe intellectual disability, hypoplasia/aplasia of the nails of the thumb and great toe, and pseudo-myopathic appearance^5–14^.

Apart from their physiological functions, Kv10.1 channels are markedly up-regulated in many cancer cells such as melanomas, breast carcinomas, soft-tissue sarcoma, cervical carcinoma, neuroblastomas, gliomas, gastric and colorectal cancer, esophageal squamous cell carcinomas, and hepatocellular carcinoma^15–23^. The aforementioned finding is consistent with the notion that Kv10.1 channels facilitate cell proliferation and, thus, Kv10.1 inhibitors may be therapeutically beneficial. An intensive effort focusing on Kv10.1 has been underway to develop novel therapeutics^24,25^.

Despite the progress in characterizing the neurological and cancer phenotypes associated with dysregulation of Kv10.1 channels, our knowledge of the channel regulation mediated by endogenous modulators remains scarce. Kv10.1 channels are strongly inhibited by Ca^2+^/calmodulin^26^, reactive oxygen species^27^, and the phospholipid PIP_2_^28,29^, and there exists the possibility that more potent endogenous modulators remain to be discovered. Here, we identify heme and hemin as potent and high-affinity endogenous modulators of Kv10.1 channels.

Heme [Fe(II) protoporphyrin-IX] is a ubiquitous cofactor essential for numerous hemoproteins, such as hemoglobin, myoglobin, respiratory cytochromes, cytochrome P450 enzymes, oxidases, and catalases. Biological systems rely on hemoproteins to serve a number of important functions, for example, oxygen transport, respiration, drug detoxification, oxidative metabolism, thyroid hormone synthesis, and signal transduction^30,31^. Recent years have witnessed growing recognition of heme and its oxidized form hemin (with Fe^3+^ as central ion) as a non-genomic modulator of ion channel function. For instance, intracellular free heme acutely modulates the function of large-conductance voltage- and Ca^2+^-activated K^+^ channels (Slo1 BK_Ca_)^32^, epithelial Na^+^ channels^33^, ATP-sensitive K^+^ channels (K_ATP_)^34^, and rapid inactivation of Kv channels mediated by their N-terminal domains (K_V_1.4 K^+^ channels^35^) or by Kvβ subunits^36^. A recent report also shows that the Per-ARNT-Sim (PAS) domain of hERG3 (Kv11.3) channels harbors a binding site for heme^37^, suggesting that the channels of the EAG family, comprising Kv10–Kv12, which all have a PAS domain in the N termini of their pore-forming α subunits, might be subject to regulation by hemin.

Here, we show that Kv10.1 channels are inhibited by low nanomolar concentrations of intracellular heme and hemin and, thus, are more sensitive than the channels previously shown to be modulated by heme and/or hemin (see above). By using electrophysiological and biochemical techniques, we reveal that the impact of heme on the channel does not depend on the PAS domain but that heme directly binds to the intracellular C-linker region of the channel, where it most likely induces a conformational change to interfere with channel opening.

## Results

### Hemin potently inhibits Kv10.1 currents

**Figure 1:**
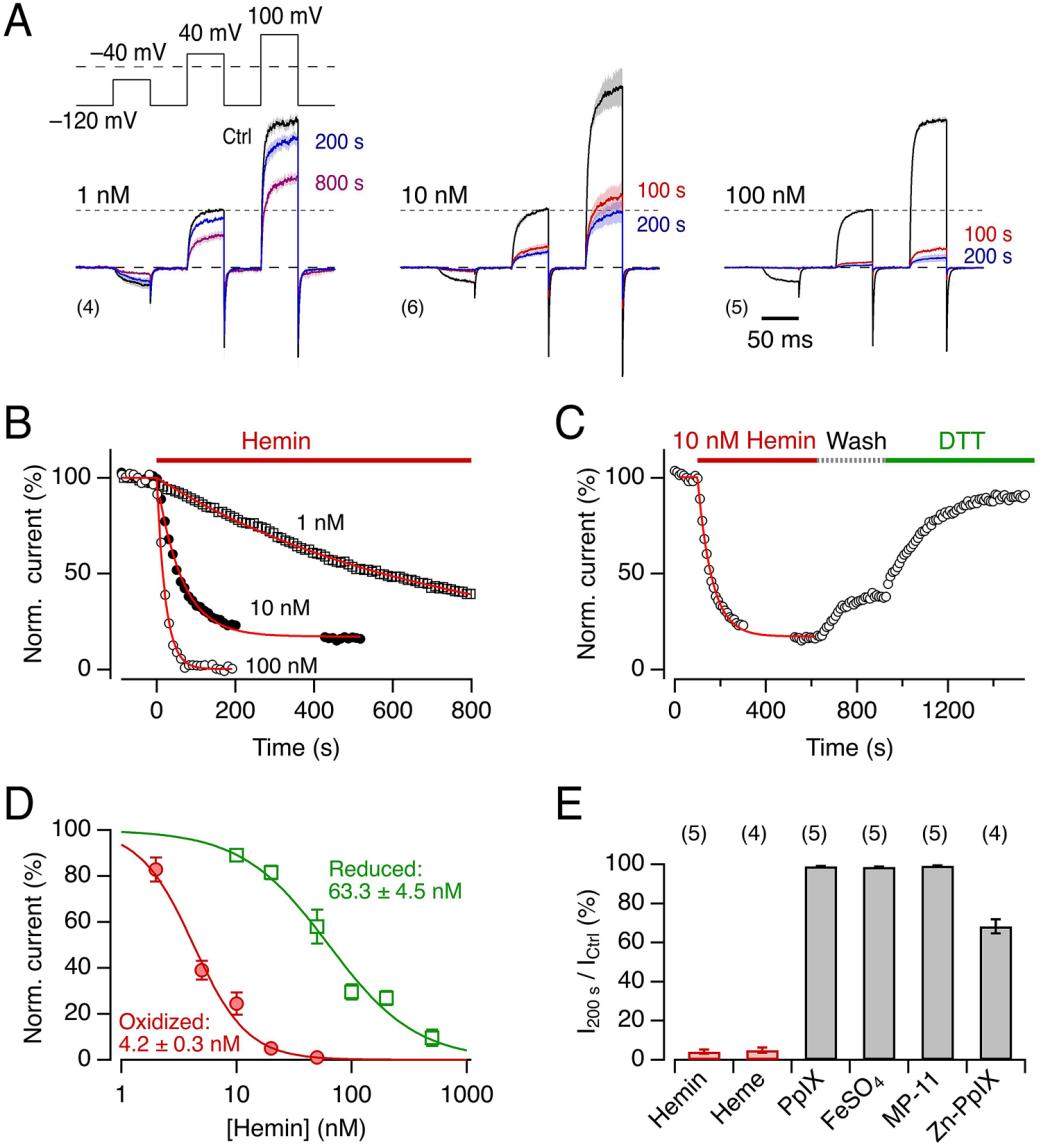
Kv10.1 current inhibition by hemin. (**A**) Mean traces of Kv10.1 channel currents from *Xenopus* oocyte inside-out patches, stimulated with the indicated pulse protocol, before (black) and recorded at the indicated times (color) after hemin application at 1 nM (left), 10 nM (center), or 100 nM (right). Traces are normalized to the maximal current at 40 mV obtained under control conditions. Thick traces are means, sem is indicated in shading, the number of recordings *n* in parentheses. (**B**) Time course of normalized maximal currents at 40 mV with application of hemin at the indicated concentration at time zero. The superimposed curves are the results of single-exponential fits. (**C**) As in (**B**) with 10 nM hemin application, wash with control solutions (Wash), and final exposure of the inside-out patch to a control solution with 1 mM DTT. The solid line is a single-exponential data fit to characterize the onset of current inhibition. (**D**) Steady-state Kv10.1 current inhibition at 40 mV, recorded from inside-out *Xenopus* oocyte membrane patches, as a function of hemin concentration in oxidizing (no GSH, red circles) and reducing (with 1 mM reduced GSH, green squares) conditions. The continuous curves are the result of Hill fits (Eq. 3) yielding the indicated half-maximal inhibition concentrations. (**E**) Mean remaining current at 40 mV in Kv10.1-containing inside-out patches after 200-s application of hemin (Fe^3+^), heme (Fe^2+^), protoporphyrin IX (PpIX), and Zn(II) protoporphyrin IX (Zn-PpIX) (all at 50 nM concentration), as well as FeSO_4_ (1 µM) and MP-11 (1 µM). Data are mean ± sem, *n* in parentheses. For current traces, see Supplementary Fig. S1. Solutions: Standard K-Asp.

To assess the sensitivity of Kv10.1 channels to intracellular hemin or heme, we expressed the channel-coding mRNA in *Xenopus* oocytes and recorded voltage-dependent currents in membrane patches large enough to give rise to macroscopic currents using the inside-out mode of the patch-clamp method. As shown in Fig. 1A, increasing concentrations of hemin (1, 10, and 100 nM) progressively diminished the current amplitudes at the test voltages applied (− 40, 40, and 100 mV) with 10 nM inhibiting the current by more than 50%. After hemin application, the current decrease followed an exponential time course, reaching an almost complete loss of current within about 1 min at 100 nM (Fig. 1B). The inhibitory effect of hemin was only very slowly reversible by wash and was accelerated by application of the reducing reagent DTT (Fig. 1C). Less than total recovery may at least in part be associated to functional decline of Kv10.1 channels in the excised patch configuration. The overall inhibitory potency was dependent on [hemin]_i_ with a half-maximal inhibitory concentration of 1.7 ± 0.3 nM at − 40 mV and 4.2 ± 0.3 nM at 40 mV and a Hill coefficient of about 1 (Fig. 1D). At 40 mV, we also measured the impact of hemin on Kv10.1 channels in the presence of reduced glutathione (GSH) to mimic the intracellular redox environment. The *IC*_50_ increased from about 4 nM to 63 nM with 1 mM GSH present (Fig. 1D). Thus, the hemin effect on Kv10.1 channels is redox dependent, potentially implicating cysteine residues.

### Effects of heme and heme-related chemicals on Kv10.1 channels

To test for the specificity of the inhibitory action of hemin, we also measured the impact of its components, Fe^2+^ (from FeSO_4_) and protoporphyrin IX (PpIX), on Kv10.1 channels. Kv10.1 current was unaffected 200 s after application of FeSO_4_ (1 µM) or PpIX (50 nM). Application of MP-11, a small peptide of microperoxidase to which heme is covalently bound, did not affect Kv10.1 channel current either (Fig. 1E), suggesting that only the free form of heme inhibits Kv10.1 channels. The reduction of the ferriheme iron to the ferrous form (Fe^2+^) by sodium dithionite (1 mM) did not alter the current inhibition efficacy, indicating that the electronic state of the iron center is not crucial. Zinc protoporphyrin IX partially inhibited the Kv10.1 current (Fig. 1E). For current traces, see Supplementary Fig. S1. These results show that only metal-ligated protoporphyrin IX in a free form diminishes the activity of Kv10.1 channels with free heme and hemin being particularly potent inhibitors.

### Hemin diminishes K^+^ currents through Kv10.1 channels in a voltage-dependent manner

**Figure 2:**
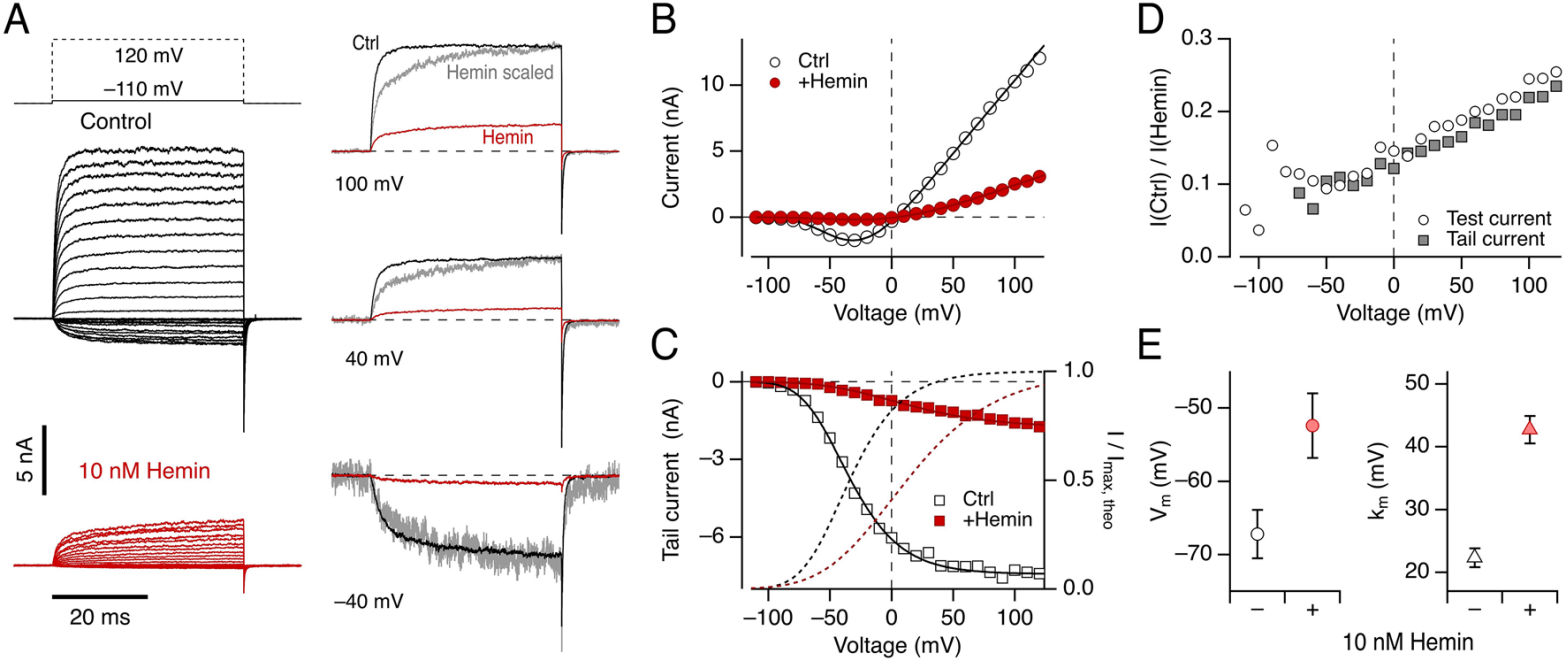
Voltage dependence of hemin-induced Kv10.1 inhibition. (**A**, left) Superposition of current traces recorded from inside-out patches according to the indicated pulse protocol in which depolarizations ranged from − 110 to 120 mV in 10-mV increments and 5-s interval before (black) and after (red) application of 10 nM hemin. Holding voltage: − 80 mV. (**A**, right) Current traces from the experiment shown on the left for the indicated voltages before (black) and after (red) hemin application. The gray traces are scaled hemin traces to match the maximal control current. Scale factors applied: 100 mV: 3.92; 40 mV: 5.2; − 40 mV: 10.7. (**B**) Mean current measured at the end of test depolarizations as a function of voltage with superimposed data fits according to Eq. (1). (**C**) Tail currents at − 140 mV as a function of test voltage with superimposed Boltzmann fits (Eq. 2). The dashed lines indicate the current normalized to the maximal current obtained in the fits (right axis). (**D**) Voltage dependence of relative current remaining after application of 10 nM hemin determined from test currents (B) (circles) and tail currents (C) (squares). (**E**) Parameters describing the voltage dependence of channel activation according to Eq. (1) without (−) and with 10 nM hemin (+); data are mean ± sem (*n* = 6). Solution: Standard K-Asp.

The inhibitory effect of hemin could be interpreted as hemin being a pore blocker of the Kv10.1 channel. To gain insight into the potential mechanism of channel modulation, we measured the voltage-dependent channel activation at 10 nM hemin, i.e., a concentration diminishing the current amplitude by more than 50% but still leaving enough current for functional analysis. Hemin slows the activation kinetics at positive voltages and accelerates the deactivation kinetics (Fig. 2A, right). Additionally, hemin shifts the half-activation voltage per subunit (*V*_m_) to the positive direction from − 67.2 ± 3.3 mV to − 52.4 ± 4.4 mV (*n* = 6) and the slope factor (*k*_m_) from 22.3 ± 1.5 mV to 42.7 ± 2.2 mV (*n* = 6) (Fig. 2B,C,E). From the relative fraction of the hemin effect on test currents (Fig. 2B) and tail currents (Fig. 2C), it is evident that the inhibitory effect is stronger at negative voltages, and the effect becomes weaker at positive voltages (Fig. 2D). The results suggest that hemin modifies the voltage dependence of Kv10.1 channel activation, and the strongest impact of hemin on the channel is expected to occur at resting voltages. It is also conceivable that the slow current component at high voltages in the presence of hemin (see Fig. 2A, scaled traces) originates from partial voltage-dependent unbinding of hemin.

### Comparison with other delayed rectifier K^+^ channels

**Figure 3:**
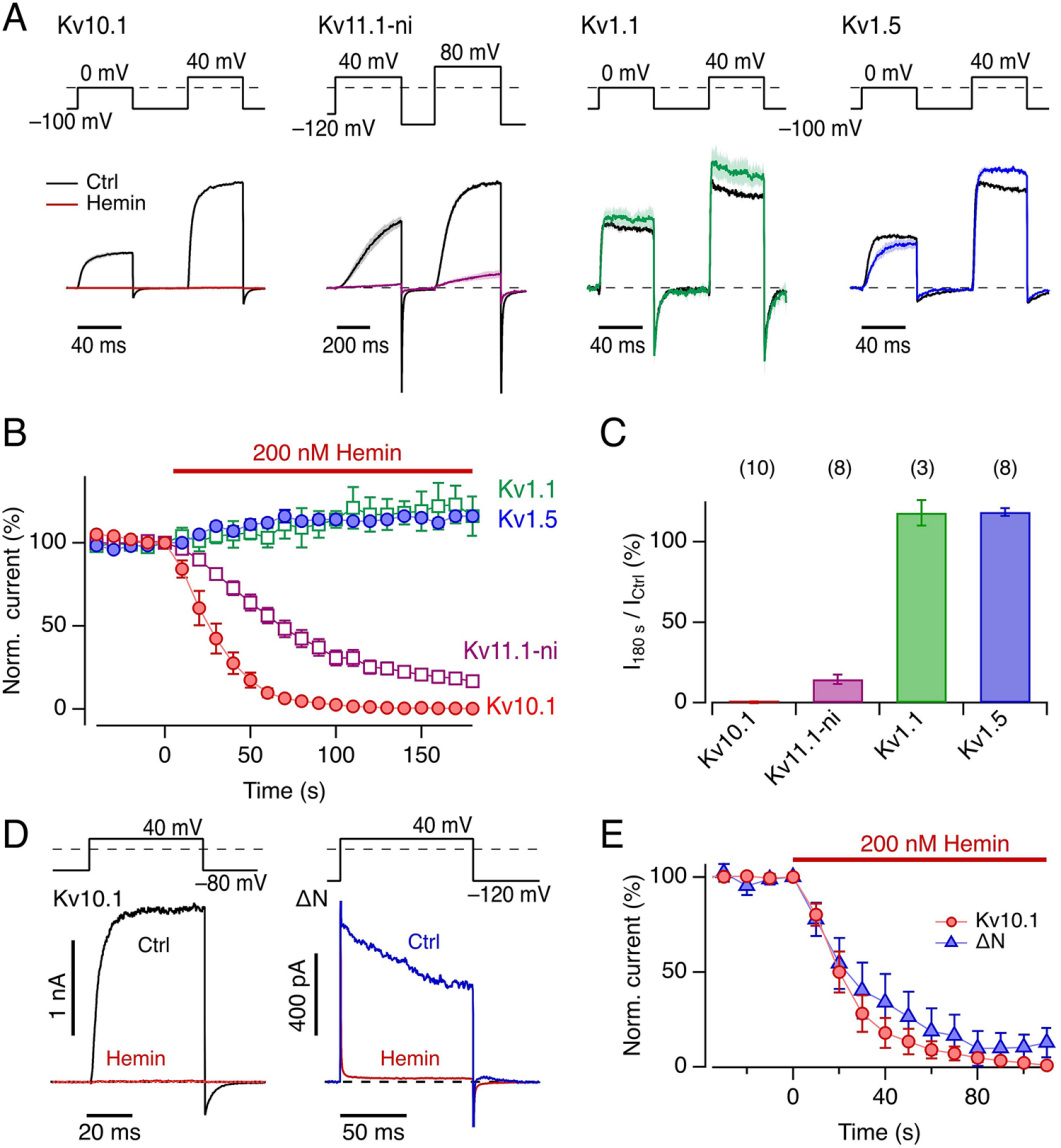
Effect of intracellular hemin on voltage-gated K^+^ channels. (**A**) Averaged current responses, normalized to the maximal control current at 40 mV (80 mV for Kv11.1-ni), of the indicated channel types recorded from inside-out patches of *Xenopus* oocytes before (black) and 180 s after (color) application of 200 nM hemin. Depolarization steps were applied to 0 and 40 mV for Kv10.1, Kv1.1, and Kv1.5 channels, and to 40 and 80 mV for Kv11.1-ni. Thick traces are means; shading indicates sem; for *n* see panel (**C**). (**B**) Normalized, mean maximal currents at 40 mV (80 mV for Kv11.1-ni) as a function of time after application of 200 nM hemin at time zero. Straight lines connect data points for clarity. (**C**) Averaged relative current remaining 180 s after application of 200 nM hemin for the indicated channel types. Data are mean ± sem, the numbers of independent experiments are indicated in parentheses. (**D**) Representative current traces recorded from inside-out *Xenopus* oocyte patches with Kv10.1 wild-type channels (left) at 40 mV before (black) and 150 s after application of 200 nM hemin. Right: Current traces for the N-terminal deletion mutant (∆N) before (blue) and 90 s after application of 200 nM hemin (red). Holding potential for Kv10.1 was − 80 mV and for Kv10.1∆N − 120 mV; the latter traces are shown without p/n leak subtraction because of high channel activity in the leak voltage range. (**E**) Time course of current decrease after application of 200 nM hemin for the indicated channel types: Kv10.1 (*n* = 8) and Kv10.1∆N (*n* = 4).

Kv10.1 channels belong to the large Kv family of depolarization-activated K^+^ channels, all of which are formed by four α subunits with similar functional modules such as voltage sensor and pore domain. To infer a potential specificity of the heme impact on Kv10.1 channel activation, we also assessed the hemin sensitivity of select Kv channel isoforms in inside-out patches from *Xenopus* oocytes with application of 200 nM hemin, i.e., a concentration that completely inhibits Kv10.1 channels after about 100 s (Fig. 3A,B). The related Kv11.1 (hERG1) channels with the pore mutation S620T to remove inactivation (Kv11.1-ni) and, hence, to make them more easily accessible to inside-out recordings from *Xenopus* oocyte membranes, were also potently inhibited by hemin, leaving only about 10% of the current after 200 s but showing a slower onset than observed for Kv10.1 channels (Fig. 3A,B). Under identical conditions, Kv1.1 and Kv1.5 channels were not inhibited by hemin (Fig. 3A–C).

In contrast with the inhibitory effect observed with application of hemin to the intracellular side, extracellular application of hemin (200 nM and 1 µM) did not decrease whole-cell currents from Kv10.1 and Kv11.1 channels expressed in HEK293T cells (Supplementary Fig. S2).

**Figure 4:**
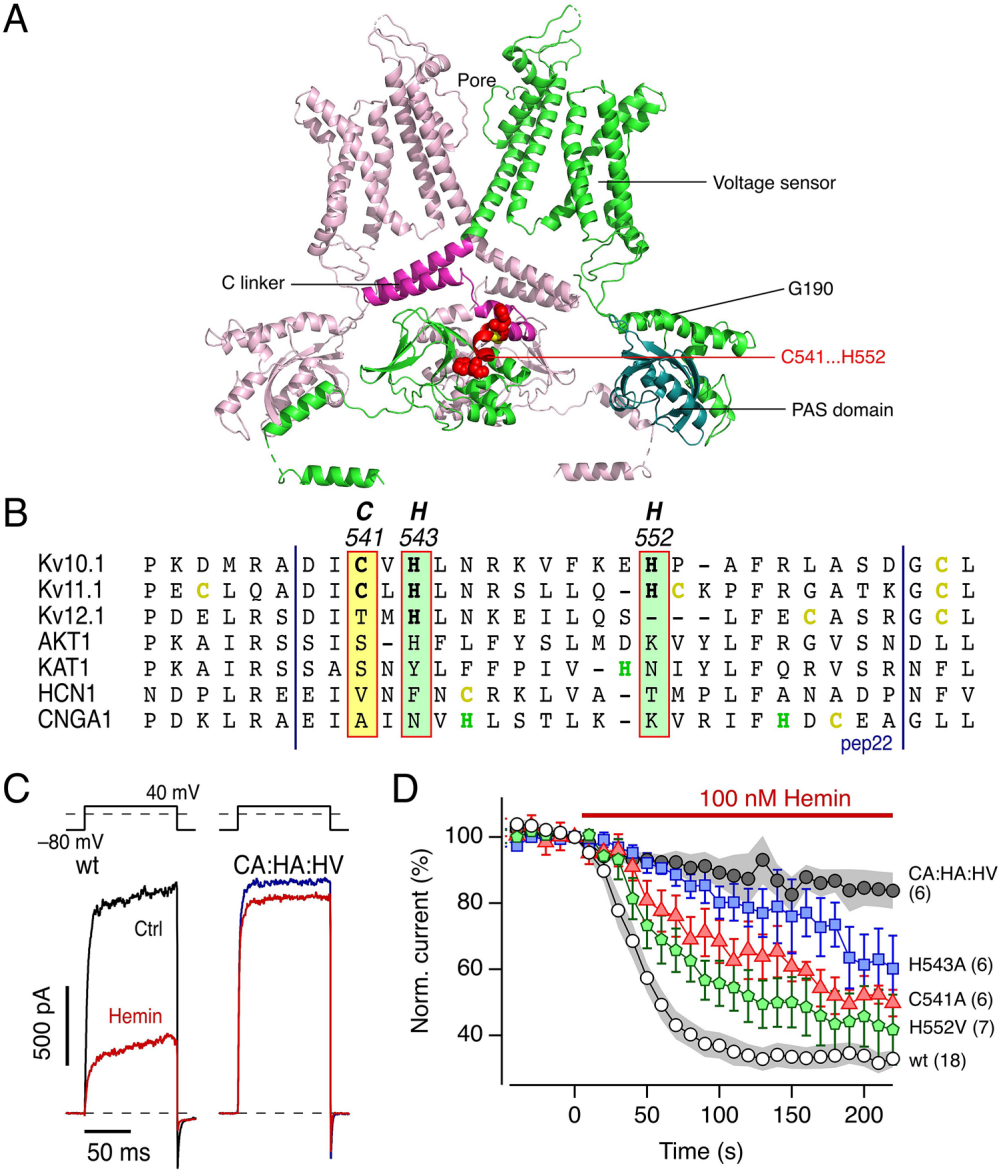
Involvement of the C-linker motif CxHx_8_H for the impact of hemin on Kv10.1. (**A**) Two opposing subunits of rat Kv10.1 (5K7L)^53^ indicating for one subunit (green) the transmembrane segments with the voltage sensor, the N-terminal PAS domain (cyan) and the deletion site G190 to yield Δ2-190, as well as the C-terminal C linker (magenta) which ends in the identified hemin-binding motif encompassing C541..H552 (red). For clarity, the other two subunits in the view direction as well as the calmodulin molecules present in 5K7L are not shown. (**B**) Multiple sequence alignment of the C-linker region of the CNBD family of channels shows that the heme-binding motif (CxHx_8_H) is conserved in some members of the EAG family (Kv10.1, Kv11.1), while absent in ELK1 (Kv12.1), in the plant K^+^ channels AKT1 and KAT1, and the cyclic nucleotide-gated channels HCN1 and CNGA2. The putative heme-binding motif is highlighted. The vertical blue lines mark the peptide sequence used for the synthesis of 22-mer peptides. (**C**) Representative inside-out current traces from *Xenopus* oocyte membrane patches at 40 mV before (black, blue) and after hemin application (100 nM in 1 mM reduced GSH, red) for Kv10.1 wild type (wt) and variant C541A:H543A:H552V (CA:HA:HV). (**D**) Time course of current reduction upon application of 100 nM hemin in reduced GSH for the indicated channel types. Data are mean ± sem with *n* in parentheses (for wt and CA:HA:HV sem in shading). Straight lines connect data points for clarity.

The hemin sensitivity of Kv10.1 and Kv11.1 might be similar to that of Kv11.3 (*KCNH*7, hERG3), another EAG family member, for which a heme-binding site has been reported in the PAS domain of the cytosolic N termini of the α subunits^37^. We therefore generated a mutant of Kv10.1 that completely lacked the N terminus (ΔN, Δ2–190), thus comprising no PAS domain (Fig. 4A). Current carried by this mutant in inside-out patches—similarly to the wild type—was completely inhibited by application of 200 nM hemin (Fig. 3D,E), thus rejecting the idea that heme binding to the PAS domain causes the Kv10.1 channel’s heme sensitivity. It should be noted that variant ΔN has altered gating properties, requiring strong hyperpolarization to keep the channel closed.

### Identification of the molecular loci required for the hemin–Kv10.1 channel interaction

With the observation of the potent inhibition of the Kv10.1 channels by hemin and the exclusion of the PAS domain as a possible binding site, we sought to identify the molecular locus of Kv10.1 that mediates this interaction. Amino-acid sequence inspection reveals that Kv10.1 channels do not contain the heme-binding motif “CxxCH” as found in cytochrome c and Slo1 BK_Ca_ channels^32^. However, there is a potential heme-binding motif CxHx_8_H, with a cysteine residue (C541) and two histidine residues (H543 and H552), in the C-linker region of the channel protein (Fig. 4A,B), connecting the last transmembrane segment (S6) with the large intracellular cytosolic domain. Sequence alignment of the C-linker region of the cyclic nucleotide-binding domain (CNBD) family of channels comprising of EAG family (Kv10.1, Kv11.1, Kv11.3, Kv12.1), plant K^+^ channels (AKT1 and KAT1), and cyclic nucleotide-gated channels (HCN1 and CNGA2) revealed the conservation of this potential heme-binding motif CxHx_8_H only in the EAG family (modified to CxHx_8_C in Kv11.3), while absent in plant K^+^ and CNG-gated channels (Fig. 4B). Consistent with the absence of this motif in CNGA2, these channels in inside-out patches from *Xenopus* oocytes were insensitive to 200 nM hemin applied to the intracellular side (Supplementary Fig. S3).

We tested the Kv10.1 variants C541A, H543A, and H552V for hemin inhibition (the variant H552A did not result in functional channels). Application of 100 nM hemin under reducing conditions to the intracellular side of inside-out patches expressing these singly mutated channels showed diminished but clearly observable inhibition (Fig. 4D). The combination of these mutations together, without noticeable influence on channel gating (Fig. 4C), largely eliminated the hemin inhibitory effect (Fig. 4C,D); C541, H543, and H552 are therefore critical residues on Kv10.1 for hemin inhibition.

### Hemin binding to peptides and recombinant channel fragments harboring a CxHx_8_H motif

**Figure 5:**
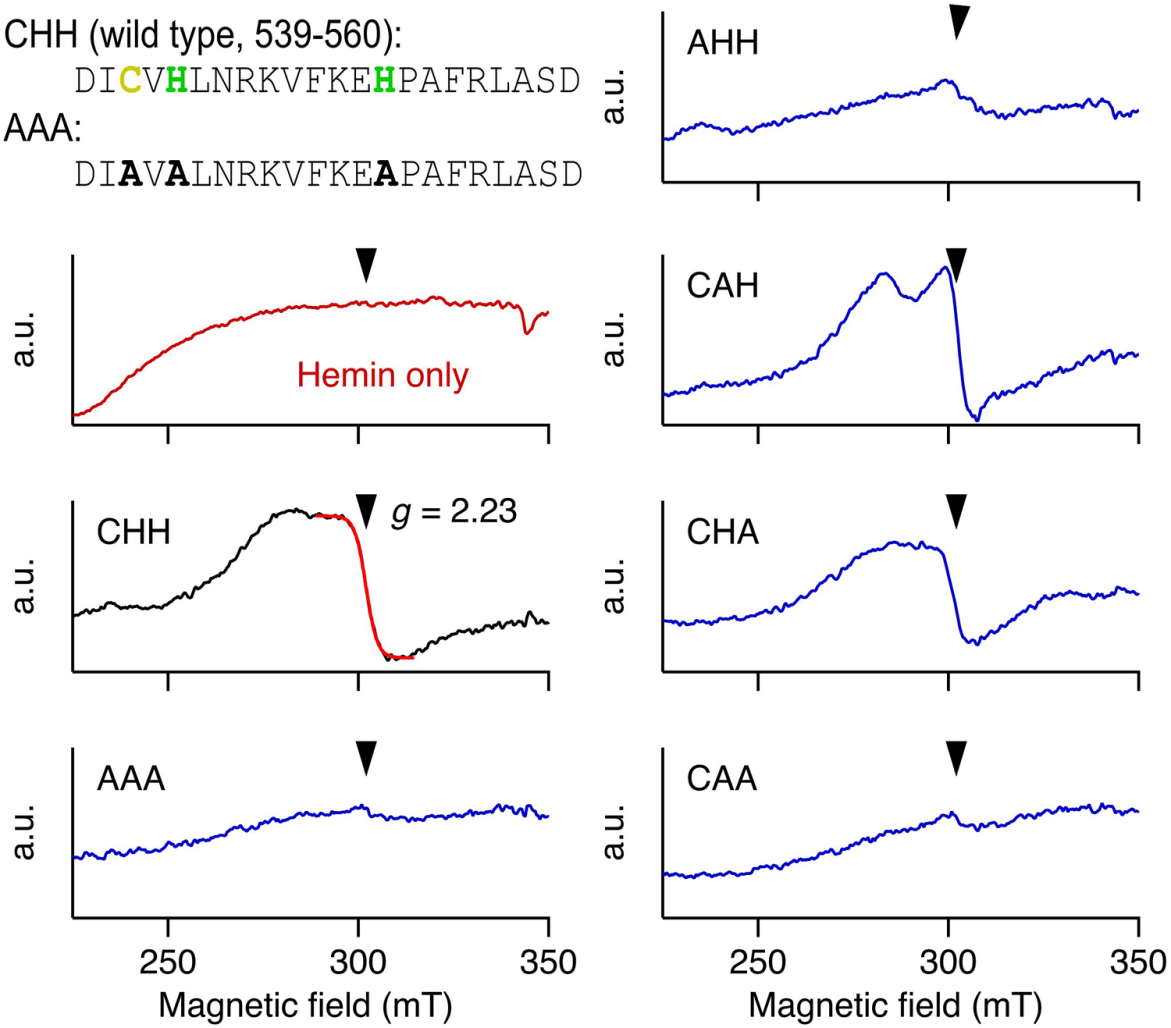
Hemin binding to Kv10.1 C-linker peptides confirmed with EPR spectroscopy. EPR measurements of 22-mer C-linker peptides encompassing the heme-binding residues C541, H543, and H552 of the indicated peptides (125 µM) and hemin (100 µM) at 9 K. Arrows mark the *g* factor 2.23, representing rhombic signals typical for axial ligation of the low-spin hemin iron. Abbreviations used: (wt) CHH, (C541A:H543A:H552A) AAA, (C541A) AHH, (H543A) CAH, (H552A) CHA, and (H543A:H552A) CAA. (top, left) Sequences for the wild-type (CHH) and mutant AAA peptide.

The results presented so far suggest that hemin physically binds to the heme-binding motif CxHx_8_H in the C-linker region of the Kv10.1 channel. To evaluate the interaction quantitatively, we employed electron paramagnetic resonance (EPR) spectroscopy with 22-mer peptides corresponding to the C-linker segment including the CxHx_8_H motif and variants thereof. When incubated with hemin, the wild-type peptide (termed CHH) showed a rhombic signal typical for low-spin Fe^3+^ with Landé factor *g* = 2.23 (Fig. 5), which is almost identical with our previous report on hemin-Kv1.4 N-terminal peptide interaction^35^. Importantly, cysteine variant C541A (termed AHH), the double-histidine variant H543A:H552A (termed CAA), and a combination of the three mutations C541A:H543A:H552A (termed AAA) abolished the rhombic signal, while the spectra of the single histidine variants H543A (CAH) and H552A (CHA) had some similarity with that of the wild type. The cysteine and both histidines in the CxHx_8_H motif thus appear to be relevant for hemin binding to the Kv10.1 channel.

A compatible ranking of the binding capacity was obtained for the analysis of variants of CxHx_8_H motif 22-mer peptides incubated with increasing hemin concentrations using UV/Vis spectroscopy in the range of 300–600 nm (Supplementary Fig. S4). For the peptides studied, a bathochromic shift of the Soret band to 416–424 nm was observed, and noticeable differences were detected for the corresponding *K*_D_ values. While the wild-type 22-mer showed strong binding affinity (1.06 ± 0.93 µM) and a Soret shift to 416 nm, single substitutions of histidine residues in peptides CAH and CHV lowered the binding affinity (CAH: 4.57 ± 0.91 µM, CHV: 5.8 ± 2.7 µM). Interestingly, a shift of the Soret band deviating from the wild-type 22-mer was observed for the CHV variant (424 nm). Mutation of both histidines (CAV) showed a comparable shift to 423 nm concomitant with a further decrease in binding affinity (28.0 ± 6.8 µM) approximating a non-binding interaction of heme to the peptide. Analysis of variant C541A (AHH) also revealed an increased *K*_D_ value (9.5 ± 2.9 µM) and a Soret shift to 417 nm, indicating that all three amino acids are involved in binding to hemin.

The synthetic peptide approach may not fully recapitulate the hemin–channel interaction. We thus recombinantly produced and purified the C-linker/CNBHD protein comprised of 196 amino acids from Kv10.1:M478-N673 fused to MBP-(His)_6_ termed as “pep196” carrying the CxHx_8_H heme-binding motif. When analyzed with microscale thermophoresis (MST), a method allowing to work with substantially smaller hemin concentrations than required for UV/Vis spectroscopy, titration of hemin against labelled Kv10.1 pep196 induced a change in the MST signal (Supplementary Fig. S5), which reflects temperature-dependent sample mobility. Determination of the binding strength revealed tight binding with a binding constant of 150 ± 68 nM. Concentrations of hemin greater than 625 nM resulted in fluorescence quenching suggesting unspecific binding of hemin or binding to the His tag. In the low-concentration range a much weaker MST signal was recorded for variant C541A:H543A:H552V, supporting the heme-binding capacity of the C-linker domain (Supplementary Fig. S5).

## Discussion

The results described here, obtained with electrophysiological and various binding assays, together demonstrate that Kv10.1 channels are directly inhibited by free hemin with an exquisite sensitivity with a half-inhibitory concentration of ~ 4 nM. This high affinity is in contrast with the lower affinities of similar modulation phenomena previously reported for other ion channel types: Slo1 BK_Ca_ (*IC*_50_ ~ 70 nM^32^), ENaC (*IC*_50_ ~ 24 nM^33^), and Kv1.4 (*EC*_50_ ~ 20 nM^35^). The high-affinity Kv10.1–hemin interaction suggests that intracellular heme may play yet-to-be discovered physiological roles in the cells expressing Kv10.1 channels.

Hampered by various technical constraints, the cytosolic free heme concentrations in mammalian cells are yet to be determined, with estimates ranging from ~ 5 to ~ 150 nM^38–42^. In line with these estimates, the *K*_D_ values of heme oxygenase 1 (HMOX1) and 2 (HMOX2) are about 1 µM and 0.4 µM, respectively. Thus, Kv10.1 channels with an *IC*_50_ of ~ 4 nM hemin or about 60 nM under reducing conditions possess great potential for interactions with heme/hemin and suggest that Kv10.1 channels may have heme/hemin bound under physiological conditions although we did not see a clear indication that some of the channels were inhibited by endogenous heme in *Xenopus* oocytes before exogenous application. It is also possible that the presence of GSH not only affects the redox properties but also scavenges free hemin by binding^43^. With respect to the remarkably low *IC*_50_ of Kv10.1 channels to hemin under ambient conditions (~ 4 nM), it is important to note that the actual redox state of heme/hemin in the cytosol is not really clear. Given the overall reducing conditions in the mammalian cell, the predominant presence of heme with ferrous iron (Fe^2+^) was commonly assumed. However, recent evidence based on heme oxygenase-2 (HO-2) activity in HEK293 cells is more consistent with labile (free) heme being oxidized^44^. Therefore, even the lowest estimate for free cytosolic heme/hemin concentration (5 nM) implies that Kv10.1 channels experience heme modulation in mammalian cells. The exact contribution of the hemin modulation of Kv10.1 channels to cell physiology and pathophysiology remains to be revealed. EAG (Kv10-12) K^+^ channels contribute to neuronal and cardiac action potentials, and their failure or gain-of-function has been associated with severe cardiac and neurological diseases. Kv10.1 channels are implicated in tumor progression^17^ and early steps in development^7^, and therefore the level of available heme in cells may be a relevant factor in critical transitions of cells during cell-cycle progression or differentiation.

The EAG ion channel family, comprised of EAG (Kv10), ERG (Kv11), and ELK (Kv12), is a member of CNBD super-family of channels including hyperpolarization-activated channels, cyclic nucleotide-gated channels, and numerous plant and bacterial channels^45^. These channels share a common transmembrane core structure as well as the C-terminal CNBD and the C-linker domain that links the CNBD to the transmembrane domain. Alignment of the C-linker region of the CNBD family of channels revealed a conserved heme-binding motif (C541, H543, and H552) in Kv11.1 channels, while the plant K^+^ channels KAT1 and AKT1 carry no cysteine and only one single histidine (Fig. 4B). CNGA2 and HCN1 channels harbor one cysteine and one histidine in the C-linker region (Fig. 4B), but our electrophysiological results show that CNGA2 channels are insensitive to hemin (Supplementary Fig. S3). This observation indicates that the hemin sensitivity is specific to the C-linker cysteine and histidine residues in Kv10.1 and Kv11.1 channels.

Previous studies have shown that heme can bind to the PAS domains of various proteins^46^. Since the N terminus of Kv10.1 also harbors a PAS domain, we functionally examined a deletion construct that lacks the PAS domain. Even after deleting 189 residues in the N terminus (Kv10.1∆N), this channel fully retains its sensitivity to intracellular hemin (200 nM; Fig. 3D,E). Although the gating properties of Kv10.1∆N are altered with respect to the wild type, this finding demonstrates that the PAS domain of the Kv10.1 channel is not responsible for the hemin effect described in this study. This conclusion is in contrast with the suggestion by Burton et al*.*^37^ that the PAS domain mediates the inhibitory effect of heme on Kv11.3 (hERG3). Burton et al*.*^37^ observed the hemin effect on Kv11.3 channels from the intracellular and the extracellular side of the channel. Kv11.3 channels were about half-inhibited by 500 nM hemin^37^, while we observed that the half-maximal inhibitory concentrations for Kv10.1 were 4.2 nM under ambient and 63 nM under reducing conditions. Extracellular application of even 1 µM hemin in HEK293T cells neither diminished the current amplitude of Kv10.1 nor of Kv11.1 (Supplementary Fig. S2). Structurally, Kv10.1, Kv11.1, and Kv11.3 share similar C-linker and N-terminal PAS domains but multiple mechanisms may be operative to account for the above divergent observations. Our mutagenesis using Kv10.1 strongly suggests that the inhibitory effect of hemin does not require the N-terminal PAS domain.

Kinetic analysis of the inhibitory interaction between the Kv10.1 channel and hemin at various voltages and hemin concentrations revealed that the "on" kinetics is concentration dependent, becoming faster with increasing hemin concentrations, whereas the "off" kinetics is very slow and concentration independent. We suggest that a single hemin molecule bound to a tetrameric channel complex has a functional impact, but we cannot exclude multiple—in particular low-affinity—binding sites within a subunit. Hemin is a gating modifier rather than a pore blocker for BK_Ca_ channel function, increasing the open probability (*P*_o_) at negative voltages and decreasing it at more positive voltages^47^. Hemin also dramatically lowers the *P*_o_ in ENaC channels^33^. Our results also indicate that hemin modifies conformational changes involved in Kv10.1 channel gating: hemin shifted the voltage of half-maximal activation by 15 mV, enhancing the inhibition at resting voltages and diminishing it under strong depolarization. Additionally, hemin delays the activation kinetics at positive voltages and accelerates the deactivation kinetics at negative voltages. Hemin also decreases the number of Kv10.1 channels available to open on depolarization so that the peak current size at very positive voltages is smaller (Figs. 1, 2).

Our previous studies on heme modulation of Slo1 BK_Ca_, Kv1.4, and Kv/Kvβ channels^32,35,36^ suggest that the hemin effect is dependent on cysteine residues. For Kv10.1 we identified a heme-binding motif containing a cysteine residue (C541), which may explain that the reducing agent DTT accelerates recovery from the hemin effect (Fig. 1C) and that GSH increases the apparent inhibitory constant (Fig. 1D). The partial recovery after hemin washout may furthermore be related to hemin-mediated oxidation of the channel protein at other sites because Kv10.1 channels are strongly sensitive to select cysteine-specific modifiers^27^.

In summary, heme and hemin have a potent inhibitory gating impact on Kv10.1 potassium channels, mediated by the cysteine- and histidine-containing binding motif (CxHx_8_H) in the C linker connecting the transmembrane domain and cytoplasmic CNBD. The location of this motif in the C-linker domain is consistent with the previously postulated notion that the C-linker domain is a critical modulator of EAG channel gating^27,48^. Given the well-recognized importance of Kv10.1 channels in a wide variety of pathophysiological conditions, the identification of heme and hemin as very potent natural inhibitors of Kv10.1 channels may serve as an important lead for the development of drugs targeting symptoms of Kv10.1-based gain-of-function mutations.

## Materials and methods

### Channel constructs and mRNA synthesis

We used *KCNH*1 (hEAG1, Kv10.1, acc. no. AJ0013668), *KCNH*2 (hERG1, Kv11.1, acc. no. NM_000238) and its pore mutant S620T, which exhibits strongly impaired inactivation^49^ (*KCNH*2-S620T, here referred to as Kv11.1-ni), *Kcna*1 (rat Kv1.1, acc. no. P10499), and *KCNA*5 (human Kv1.5, acc. no. P22460). Kv10.1 channel mutant constructs C541A, H543A, H552V, and Δ2–190 were prepared by using overlap-extension mutagenesis as described previously^26^. We also utilized the human cyclic-nucleotide gated channel *CNGA*2 (acc. no. NM_005140.1).

For the *Xenopus oocyte* expression system, channel constructs were cloned into the pGEM-HE plasmid, which was then used for synthesis of capped mRNA by using the mMessage mMachine kit (Ambion, Austin, TX, USA). For expression in human embryonic kidney cells (HEK293T, ATCC: CRL-1573), the channel constructs were inserted into pcDNA3.1.

### Chemicals

Hemin (Fe(III) protoporphyrin IX), protoporphyrin IX, Zn(II) protoporphyrin IX, FeSO_4_, microperoxidase (MP-11), and sodium dithionite were purchased from Sigma-Aldrich. For each electrophysiological recording, working solutions were freshly prepared by diluting the stock solution (1 mM in 30 mM KOH) with the intracellular recording solutions. The solutions were kept at 4 °C and utilized within 20 min.

### Oocyte preparation and mRNA injection

Oocytes were surgically extracted from ovarian tissue of *Xenopus* laevis that had been anesthetized by immersion in ice water/tricaine. The protocol was approved by the University Hospital Jena animal welfare committee (registration no. 02-104/13). All methods were carried out in accordance with relevant guidelines and regulations and reported in accordance with ARRIVE guidelines. Defolliculated stage V and VI oocytes were microinjected with 50 nl of mRNA coding wild-type or mutant channels and patch-clamp recordings were performed 2–4 days following mRNA injection after removing vitelline membranes.

### Electrophysiological recordings

Ion currents from *Xenopus* oocytes were measured by using an EPC-9 patch-clamp amplifier and PatchMaster software (both HEKA Elektronik, Lambrecht, Germany) at room temperature (20–24 °C). Macroscopic currents in the inside-out configuration were measured with aluminum silicate glass pipettes of about 1 MΩ resistance in the recording solutions. Modulator-containing solutions were applied directly to the pipette containing the membrane patch using a self-made multi-channel perfusion system. Solution exchange was achieved by moving the pipette tip with an inside-out patch from one outlet to the next, thus ensuring absolutely clean solutions. The intracellular solutions contained (in mM): 100 K-aspartate, 15 KCl, 1 GSH (reduced glutathione), 10 EGTA (ethyleneglycol-bis(β-aminoethyl)-*N*,*N*,*N*ʹ,*N*ʹ-tetraacetic acid), and 10 HEPES (4-(2-hydroxyethyl)-1-piperazineethanesulfonic acid) (pH 8.0 with KOH). The extracellular solution contained (in mM): 103.6 Na-aspartate, 11.4 KCl, 1.8 CaCl_2_, and 10 HEPES (pH 7.2 with NaOH). For recordings with symmetrical K^+^ concentration (termed “Standard K-Asp”), K-aspartate replaced Na-aspartate in the external solution, and the pH was adjusted with KOH. The holding potential was − 100 mV, and depolarizing pulses were applied with a sweep interval of 2 s for current–voltage relationships and 10 s for assaying the time course of the hemin impact. Deviations are explicitly indicated.

Ion currents from HEK293T cells were recorded using the whole-cell configuration at room temperature (20–24 °C). Patch pipettes were fabricated from borosilicate glass (BioMedical Instruments, Zöllnitz, Germany) and were coated with dental wax (Patterson Dental, Mendota Heights, MN, USA) to decrease their capacitance. After fire-polishing the pipettes, resistances of 0.9–2.0 MΩ were obtained. An agar bridge connected the bath solution and the ground electrode. Up to 85% of the series resistance was electronically compensated, and all voltages were corrected for liquid junction potential. For Kv10.1 channels, the external solution composed of (in mM) 146 NaCl, 4 KCl, 0.2 CaCl_2_, 0.2 MgCl_2_, and 10 HEPES (pH 7.4 with NaOH) and the internal solution contained (in mM) 115 K-Asp, 15 KCl, 2.56 MgCl_2_, 10 EGTA, and 10 HEPES (pH 7.4 KOH). For Kv11.1 channels, the external solution (in mM): 140 NaCl, 10 KCl, 2 CaCl_2_, 2 MgCl_2_, 10 HEPES (pH 7.4 with NaOH) and the internal solution (in mM): 130 KCl, 2.56 MgCl_2_, 10 EGTA, 10 HEPES (pH 7.4 with KOH) were used. Working solutions with hemin were applied by adding 1 ml of 3 × concentrated hemin solution to the bath chamber with 2 ml control solution.

### Expression and purification of Kv10.1 C-terminal peptides

The amino-acid sequence M478-N673 of Kv10.1 encompassing the heme-binding site was subcloned into a modified pMALc2T vector carrying maltose-binding protein (MBP) and a (His)_6_-tag, thus generating an MBP-(His)_6_-peptide fusion protein. The recombinant protein was expressed in *Escherichia coli* BL21(*DE3*) pRIL. Cells were grown in YT medium and were induced with 0.5 mM IPTG at 22 °C for 20 h. Cells were harvested by centrifugation at 4000*g* for 20 min at 4 °C. The protein pellet was solubilized in buffer A (50 mM Tris, 500 mM NaCl, 5 mM DTT, pH 8.0) and lysed by sonification. The cell lysate was then centrifuged and the clear supernatant was used in the final purification system consisting of HisTrap FF crude column (GE Healthcare), washed with buffer A + 16 mM imidazole, and eluted with buffer B (buffer A + 250 mM imidazole). The eluted fractions containing the His-tagged Kv10.1 M478-N673 were further purified using a gel filtration column (Superdex^®^ 200 Increase 10/300 GL) equilibrated with buffer C (PBS, pH 7.4, 1 mM TCEP).

### Microscale thermophoresis (MST)

MST experiments were performed with the Monolith NT.115 (NanoTemper Technologies, Munich, Germany) in a buffer containing PBS with pH 7.4, 2 mM GSH, and 0.05% Tween-20. Purified Kv10.1 was labeled according to the manufacturer´s instructions using the Labelling Kit Monolith NT RED-NHS (NanoTemper Technologies), and the concentration was adjusted to approximately 100 nM. Hemin was titrated in 1:1 dilutions, starting with a concentration of 40 µM. Each sample was centrifuged at 13,000 rpm for 5 min to remove aggregates before filled into hydrophilic or premium capillaries (NanoTemper Technologies).

### Peptide synthesis and purification

Peptides of 22 residues (Supplementary Table 1) were synthesized on an automated peptide synthesizer EPS 221 (Intavis Bioanalytical Instruments AG, Cologne, Germany) according to a standard Fmoc-protocol as described earlier^50^. The 22-mer control peptide “CHH” was synthesized manually (see below). The polymer support was Tentagel amide resin with a loading capacity of 0.53 mmol/g (22-mer peptides; IRIS Biotech, Marktredwitz, Germany) or Rink amide MBHA resin with a loading capacity of 0.73 mmol/g (hEAG-1; Novabiochem, Merck Biosciences, Germany).

For manual peptide synthesis, coupling reactions were performed as double couplings using Fmoc-amino acids (4 equiv.) activated with 2-(1H-benzotriazole-1-yl)-1,1,3,3-tetramethyluronium hexafluorophosphonate (HBTU, 4 equiv.), and 1-hydroxy-1H-benzotriazole (HOBt, 4 equiv.) in the presence of DMF and DIEA (8 equiv.). Fmoc removal was carried out by treating the resin twice with 20% piperidine in DMF. All deprotection and coupling steps were followed by intensive washings using DMF and DCM, alternately. Peptide cleavage and purification were performed as described earlier^50^. Analytical data are provided in Supplementary Table 1.

### UV/visible (UV/Vis) spectroscopy

Hemin (1 mM) was prepared by dissolving it in 30 mM NaOH and incubated for 30 min. Subsequently, it was diluted to 100 µM in 0.1 M HEPES buffer (pH 7.0) and directly used for titration of the peptide solution. Peptide solutions (1 mM) were prepared freshly from the lyophilized powder by addition of 0.1 M HEPES buffer (pH 7.0). Varying concentrations of hemin (0.4–40 µM) were mixed with the peptide (5–30 µM) and incubated for 30 min before measurement of the absorption spectra (λ = 230 nm to 750 nm) using a UV/Vis spectrophotometer V650 (Jasco, Gross-Umstadt, Germany), a UV-3100PC (VWR, Darmstadt, Germany), or a Multiskan Go (Thermo Fisher Scientific Inc., Waltham, MA, USA). Evaluation of the obtained spectra was performed as described earlier^51,52^.

### Electron paramagnetic resonance (EPR) spectroscopy

EPR analysis of hemin binding to 22-mer Kv10.1 C-linker peptides was performed as described previously^35^. Briefly, samples of the Kv10.1 C-linker 22-mer peptides of the wild-type sequence (termed “CHH”, DI**C**V**H**LNRKVFKE**H**PAFRLASD) and the respective single, double and triple mutants (Supplementary Table 1) were prepared in 20 mM Tris, pH 7.5, buffer with 40–50% (vol/vol) ethylene glycol at 25 °C. Samples (100 µl) were filled in EPR tubes and frozen in liquid nitrogen. EPR spectra were recorded at 9 K (Oxford ESR900 cryostat) on a Bruker Elexsys spectrometer operating in continuous wave mode with a modulation amplitude of 0.8 mT at a frequency of 100 kHz. Microwave power was set to 8 mW. The background signals were eliminated by subtracting control spectra from buffer samples (without protein and hemin) measured under identical conditions. The apparent *g* factors were determined using the WINEPR or XeprView programs (Bruker).

### Data analysis

Data were analyzed with FitMaster (HEKA Elektronik), FITMASTER NEXT (Multi Channel Systems MCS GmbH, Reutlingen, Germany), and IgorPro software (WaveMetrics, Lake Oswego, OR). Data are presented as mean ± sem with the number of independent measurements, *n*.

The relationship of maximal current at the end of the test pulses as a function of voltage was described assuming four independent subunits as follows:

1 $$I(V)\, = \,\Gamma \;(V - E_{rev} )\frac{1}{{\left( {1 + {\text{e}}^{{ - {{(V - V_{m} )} \mathord{\left/ {\vphantom {{(V - V_{m} )} {k_{m} }}} \right. \kern-\nulldelimiterspace} {k_{m} }}}} } \right)^{4} }}.$$

With the conductance Γ, the reversal potential *E*_rev_ (which is close to zero under symmetrical K^+^ concentration), the half-maximal activation voltage *V*_m_, and the slope factor *k*_m_ characterizing the voltage dependence of individual subunits. For the tail current (*I*_tail_), i.e., the maximal current obtained in a segment following the test segments, Eq. (1) reduces to:

2 $$I_{tail} (V)\, = \,\frac{{I_{\max } }}{{\left( {1 + {\text{e}}^{{ - {{(V - V_{m} )} \mathord{\left/ {\vphantom {{(V - V_{m} )} {k_{m} }}} \right. \kern-\nulldelimiterspace} {k_{m} }}}} } \right)^{4} }}.$$

Concentration (*c*) dependence of current inhibition was described with a Hill equation:

3 $$\frac{I(c)}{{I_{Ctrl} }}\, = \,\frac{1}{{1 + \left( {{c \mathord{\left/ {\vphantom {c {IC_{50} }}} \right. \kern-\nulldelimiterspace} {IC_{50} }}} \right)^{{n_{H} }} }},$$

with the half-inhibitory concentration *IC*_50_ and the Hill coefficient *n*_H_.

## Supplementary Information

Supplementary Information.

## Data Availability

The analyzed data supporting the conclusions of this article are included in the article and the supplementary material. Further datasets acquired during the current study are available from the corresponding author on reasonable request.

## Abbreviations

CNBD: Cyclic nucleotide binding domain
CNS: Central nervous system
DTT: Dithiothreitol
EAG: *Ether à go-go*
GSH: Reduced glutathione
Kv channel: Voltage-gated potassium channel
HRM: Heme-regulatory motif
MBP: Maltose-binding protein
MST: Microscale thermophoresis
PAS: Per-ARNT-Sim
PpIX: Protoporphyrin IX
wt: Wild type
