# Supplementary material for: Intracellular hemin is a potent inhibitor of the voltage-gated potassium channel Kv10.1
Source: Sci Rep. 2022 Aug 27;12:14645. doi: 10.1038/s41598-022-18975-2 (PMC9420133; doi:10.1038/s41598-022-18975-2)
Supplement: Supplementary file 1 — Supplementary Information. [file 41598_2022_18975_MOESM1_ESM.pdf]

Intracellular hemin is a potent inhibitor of the voltage-gated potassium channel Kv10.1

– Supplementary Material –

Nirakar Sahoo[1,2], Kefan Yang[1], Ina Coburger[1], Alisa Bernert[1], Sandip M. Swain[1#], Guido Gessner[1], Reinhard Kappl[3], Toni Kühl[4], Diana Imhof[4], Toshinori Hoshi[5], Roland Schönherr[1] & Stefan H. Heinemann[1*]

1: Center for Molecular Biomedicine, Department of Biophysics, Friedrich Schiller University Jena & Jena University Hospital, Hans-Knöll-Str. 2, D-07745 Jena, Germany

2: Department of Biology, The University of Texas Rio Grande Valley, 1201 West University Drive, Edinburg, TX 78539, USA

3: Institute of Biophysics, Saarland University, D-66421 Homburg, Germany

4: Pharmaceutical Biochemistry and Bioanalytics, Pharmaceutical Institute, University of Bonn, An der Immenburg 4, D-53121 Bonn, Germany

5: Department of Physiology, University of Pennsylvania, Philadelphia, PA 19104-6085, USA

# Present address: Department of Medicine, Duke University Durham, NC 27710, USA

*** Correspondence:**

Prof. Dr. Stefan H. Heinemann
Center for Molecular Biomedicine
Department of Biophysics
Friedrich Schiller University Jena & Jena University Hospital
Hans-Knöll-Str. 2
D-07745 Jena, Germany
Tel: ++49-3641-9 39 56 50
Fax: ++49-3641-9 39 56 52
e-Mail: Stefan.H.Heinemann@uni-jena.de

Supplementary Material

Supplementary Methods

UV/vis binding curves, i.e., the change in absorbance at the Soret peak as a function of the hemin concentration (x), were fit according to the following formalism [1]:

$$\Delta E = 0.5\, ee \left(x + n\,P + K_D - \sqrt{\left(x + n\,P + K_D\right)^2 - 4\,x\,n\,P} \right) \qquad \text{Eq. S1}$$

with the extinction coefficient of free and complexed heme *ee*, the peptide concentration P, the number of binding sites n, and the apparent binding constant K_D.

Supplementary Tables

Supplementary Table 1. Analytical data of peptides.

Peptide	Peptide sequence	M_w (M_w calc.) [g/mol]	Preparative HPLC t_r [min]	Analytical HPLC t_r [min]*
**"CHH"**	DI**C**V**H**LNRKVFKE**H**PAFRLASD	2594.35[a] (2593.36)	37.4[b]	12.06[d]
**"AHH"**	DI**A**V**H**LNRKVFKE**H**PAFRLASD	2562.38[a] (2561.40)	42.8[b]	11.44[d]
**"CAH"**	DI**C**V**A**LNRKVFKE**H**PAFRLASD	2528.34[a] (2527.34)	46.5[b]	13.13[d]
**"CHV"**	DI**C**V**H**LNRKVFKE**V**PAFRLASD	2556.38[a] (2555.37)	50.8[b]	14.51[d]
**"CAV"**	DI**C**V**A**LNRKVFKE**V**PAFRLASD	2490.35[a] (2489.35)	54.9[b]	15.81[d]
**"CHA"**	DI**C**V**H**LNRKVFKE**A**PAFRLASD	2528.47[a] (2527.34)	32.9[c]	25.84[e]
**"CAA"**	DI**C**V**A**LNRKVFKE**A**PAFRLASD	2463.41[a] (2461.32)	60.7[b]	27.22[e]
**"AAA"**	DI**A**V**A**LNRKVFKE**A**PAFRLASD	2431.36[a] (2429.34)	57.7[b]	27.23[e]

Mass spectroscopy peaks were detected as [a]$[M+H]^+$. Peptides were detected with MALDI-TOF-MS. Purification was performed using semipreparative RP-HPLC: [b]15-65% eluent B in 120 min and [c]15-75% eluent B in 120 min. For analytical RP-HPLC the following gradients were used: [d]20-50% eluent B in 30 min, [e]10-50% eluent B in 30 min. *All peptides were >95% HPLC pure.

Supplementary Figures

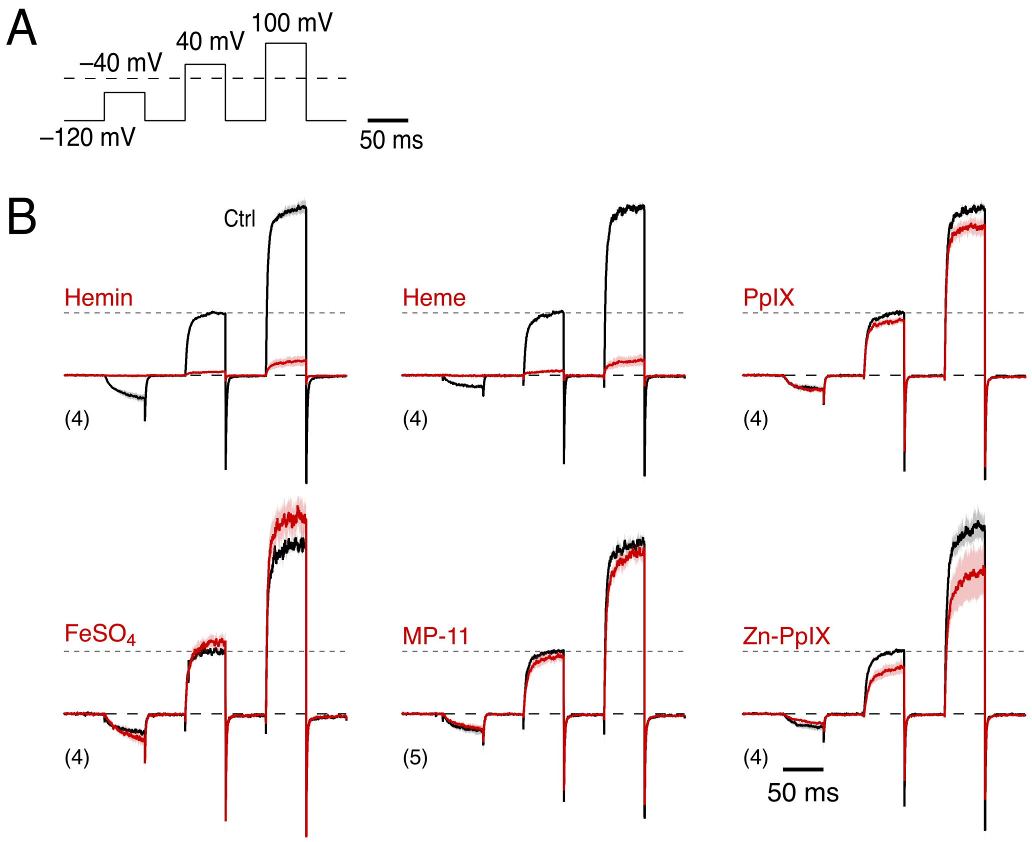

Figure S1. Impact of hemin and heme-related compounds on Kv10.1 currents. (**A**) Pulse protocol used to activate Kv10.1 channels in inside-out patches from *Xenopus* oocytes. (**B**) Mean current traces recorded before (black) and 200 s after application of the indicated compounds: hemin (Fe^{3+}, 50 nM), heme (Fe^{2+}, 50 nM), protoporphyrin IX (PpIX, 50 nM), Fe^{2+} (from 1 μM $FeSO_4$), MP-11 (1 μM), and Zn^{2+}-protoporphyrin IX (Zn-PpIX, 50 nM). Traces are normalized to the maximal current at 40 mV obtained under control conditions. Thick traces are means, sem indicated in shading, *n* in parentheses. Solutions: Standard K-Asp.

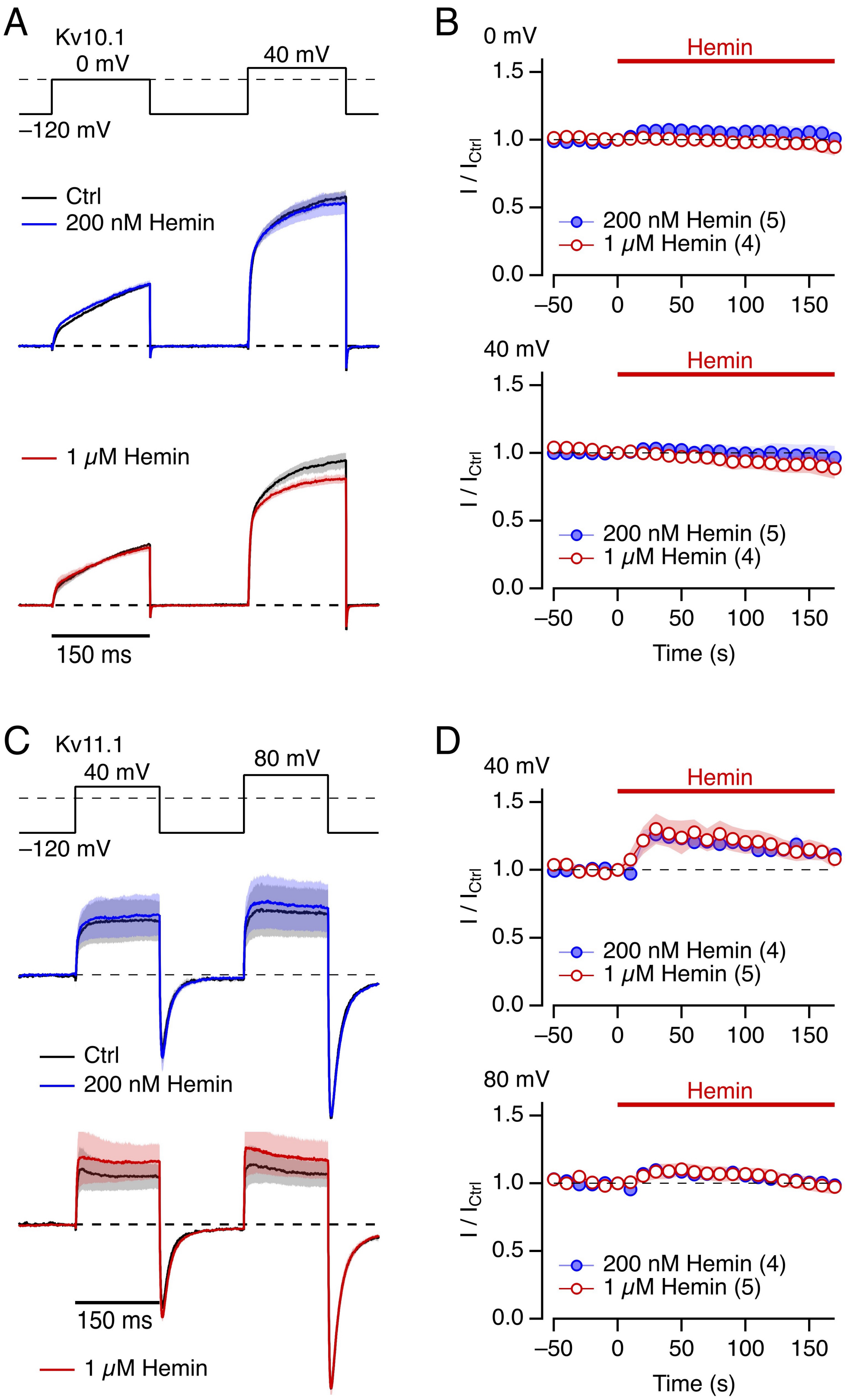

Figure S2. Whole-cell currents of Kv10.1 and Kv11.1 channels with extracellular hemin application. (**A**) Superposition of mean Kv10.1 current traces recorded from transfected HEK293T cells for the pulse protocol shown on top before (black) and after application of

hemin (color) at the indicated concentration. Thick traces are means and sem is indicated in shading (for *n* values see B). (**B**) Mean time courses of maximal outward current at 0 mV (*top*) or 40 mV (*bottom*) as a function of time after hemin application. Data are means ± sem with *n* in parentheses. (**C**) As in (A) for Kv11.1 channels. (**D**) Time courses of maximal Kv11.1 tail current at -120 mV after depolarization to 40 mV (*top*) or 80 mV (*bottom*) as a function of time after hemin application. For solutions used, see Methods

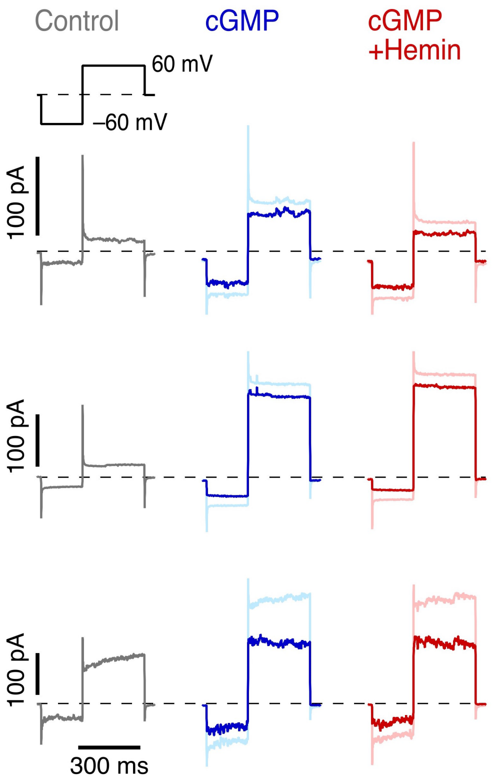

Figure S3. Cyclic nucleotide-gated channels are not inhibited by hemin. Current traces of CNGA2 channels expressed in *Xenopus* oocytes recorded from three different inside-out patches at -60 and 60 mV from a holding voltage of 0 mV under control conditions (Control, gray), after application of 50 µM cGMP (blue), and additional application of 200 nM hemin (red). Light colored traces are raw data; thick colored traces are the results after subtracting the control traces. The mean relative changes in current after hemin application were: 0.89 ± 0.20 at 60 mV and 0.94 ± 0.13 at -60 mV.

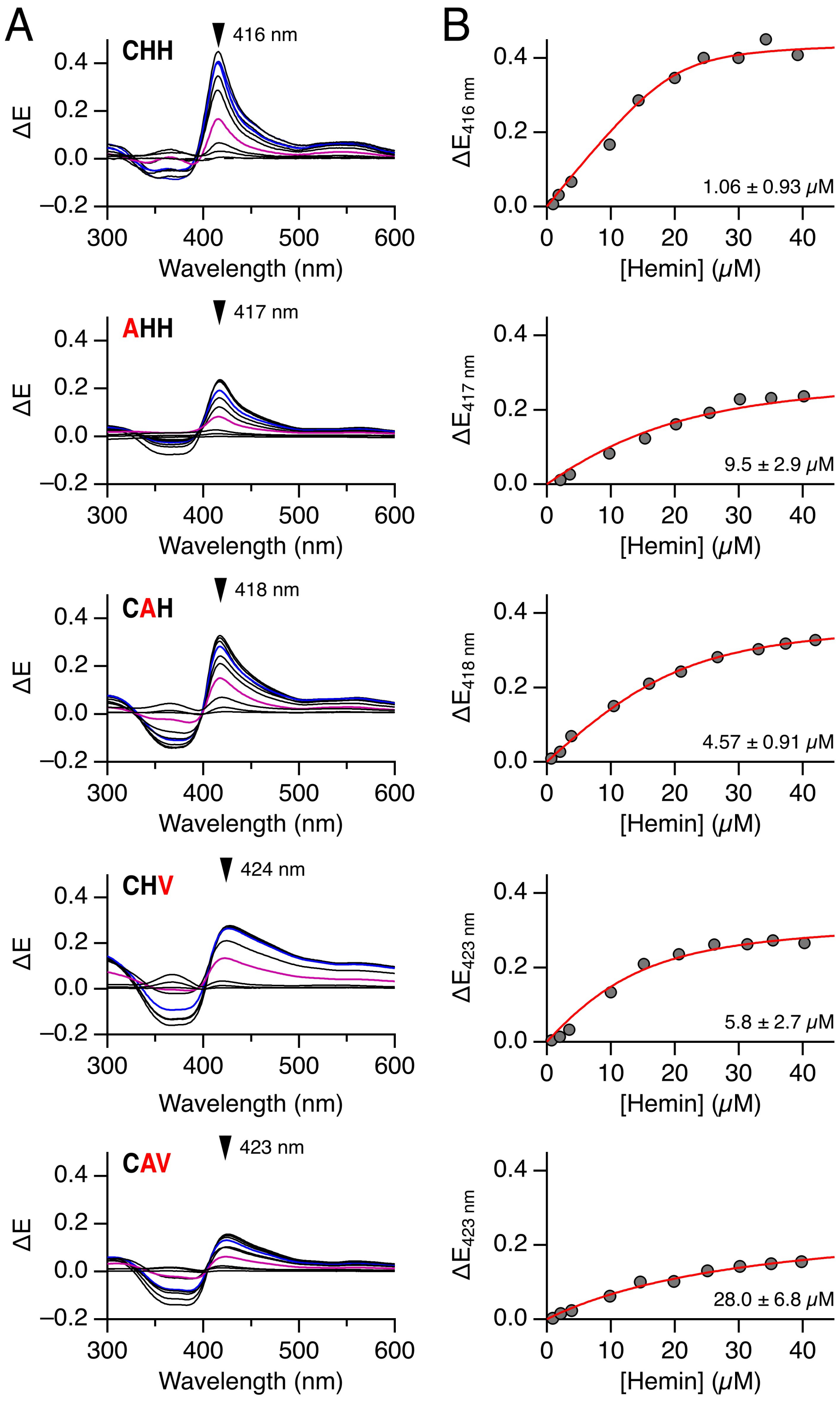

Supplementary Figure 4. Hemin binding to 22-mer Kv10.1 C-linker peptides confirmed with UV/Vis spectroscopy. UV/Vis absorbance measurements of 22-mer C-linker peptides (5-12 μM) were conducted by application of varying concentrations of heme (0-40 μM) after 30 min incubation. (**A**) Superimposed difference spectra with varying hemin concentrations for the indicated peptides. Traces in magenta denote about 10 μM and in blue 25 μM hemin.

(**B**) Peak change in absorbance at the indicated Soret peak wavelength as a function of hemin concentration. The superimposed curves are results of data fits to a binding model according to Eq. S1 assuming n = 2 for wt, AHH, and CAH, and n = 1 for CHV and CAV. The dissociation constants are given in the graphs, and the results for *ee* were: wt, 0.0222 ± 0.0016; AHH, 0.0190 ± 0.0015; CAH, 0.0193 ± 0.0007; CHV, 0.0277 ± 0.0029; CAV, 0.056 ± 0.007. An increase in the dissociation constants for binding of heme was observed for all mutated peptides compared to the wild-type peptide.

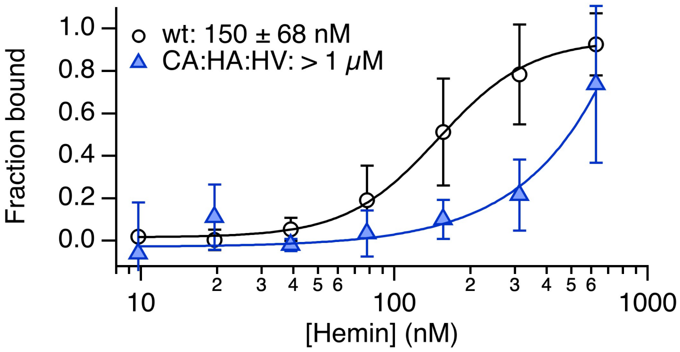

Supplementary Figure 5. Recombinant Kv10.1 C-terminal protein binds hemin. Microscale thermophoresis binding curves for the interaction of recombinant Kv10.1 MBP-His$_6$-M478-N673 polypeptide (wt) and mutant C541A:H543A:H552V (CA:HA:HV, blue) as a function of hemin concentration, normalized to the wild-type data at the highest concentration of hemin. Data are mean ± sem (n = 3; 2 protein preparations). The estimated binding constants are indicated.

References

1 Kühl, T. *et al.* Analysis of Fe(III) heme binding to cysteine-containing heme-regulatory motifs in proteins. *ACS Chem Biol* **8**, 1785-1793, doi:10.1021/cb400317x (2013).
